# Mathematical Analysis of a Prototypical Autocatalytic Reaction Network

**DOI:** 10.3390/life9020042

**Published:** 2019-05-20

**Authors:** Ekaterina V. Skorb, Sergey N. Semenov

**Affiliations:** 1 ChemBio Cluster, ITMO University, Lomonosova St. 9, Saint Petersburg 191002, Russia; skorb@corp.ifmo.ru; 2 Department of Organic Chemistry, Weizmann Institute of Science, Rehovot 76100, Israel

**Keywords:** Autocatalysis, reaction networks, origin of life, Michaelis-Menten kinetics, numerical simulations, self-replication

## Abstract

Network autocatalysis, which is autocatalysis whereby a catalyst is not directly produced in a catalytic cycle, is likely to be more common in chemistry than direct autocatalysis is. Nevertheless, the kinetics of autocatalytic networks often does not exactly follow simple quadratic or cubic rate laws and largely depends on the structure of the network. In this article, we analyzed one of the simplest and most chemically plausible autocatalytic networks where a catalytic cycle is coupled to an ancillary reaction that produces the catalyst. We analytically analyzed deviations in the kinetics of this network from its exponential growth and numerically studied the competition between two networks for common substrates. Our results showed that when quasi-steady-state approximation is applicable for at least one of the components, the deviation from the exponential growth is small. Numerical simulations showed that competition between networks results in the mutual exclusion of autocatalysts; however, the presence of a substantial noncatalytic conversion of substrates will create broad regions where autocatalysts can coexist. Thus, we should avoid the accumulation of intermediates and the noncatalytic conversion of the substrate when designing experimental systems that need autocatalysis as a source of positive feedback or as a source of evolutionary pressure.

## 1. Introduction 

Autocatalytic reactions are of profound importance for at least three problems in the chemical sciences: (i) chemical evolution and the origin of life, [1,2,3,4,5,6] (ii) dissipative chemical systems [7,8,9], and (iii) chemical signaling and information processing systems [10]. Autocatalytic self-replication is an element needed for Darwinian selection. Biological evolution is driven by cellular division and life most likely originates from prebiotic reactions that involve some form of self-replication and autocatalysis [1,11,12,13,14]. Dissipative systems, such as chemical and biological oscillators and Turing structures [15,16,17,18], often require autocatalytic reactions as a source of positive feedback. Finally, biochemical signaling systems need autocatalysis for signal amplification [10]. 

The importance of autocatalysis for determining the origin of life is twofold [19]. At the initial stages of prebiotic evolution, where organic building blocks accumulated, autocatalysis is a possible solution for the “mixtures” problem [20,21,22], which involves a low abundance of any particular reaction product from diverse starting materials because of the statistical distribution of the reaction products. Autocatalysis would accelerate the formation of a limited set of products and consume starting materials for forming these products, thus avoiding the formation of the complex mixture. At later stages of prebiotic evolution, autocatalysis serves as a driving force for the natural selection of information carriers [2,12,14,23]. It amplifies the fittest carriers against others. Many variations of information carriers have been proposed such as rybozimes [24], autocatalytic sets of polypeptides [1,2,12,14,22], or vesicles carrying compositional information [23], but autocatalysis is always a driving force behind the evolution of these species. 

Direct autocatalysis, which is a process whereby a product directly catalyzes its own production through formation of only short-living intermediates, is often differentiated from network autocatalysis, where multiple stable products cooperatively accelerate their own production [25,26,27,28,29]. Because of its high mechanistic versatility compared with direct autocatalysis, network autocatalysis has become a basis for a variety of models for determining the origin of life [1,2,11,12,14,23,30]. Nevertheless, the functional properties (e.g., the ability to evolve) of a particular network depend on their kinetic behavior which [31,32], consequently, depend on the structure of the network [33]. Therefore, it is important to analyze the kinetics of specific and chemically plausible autocatalytic reaction networks [34,35,36]. 

**Figure 1:**
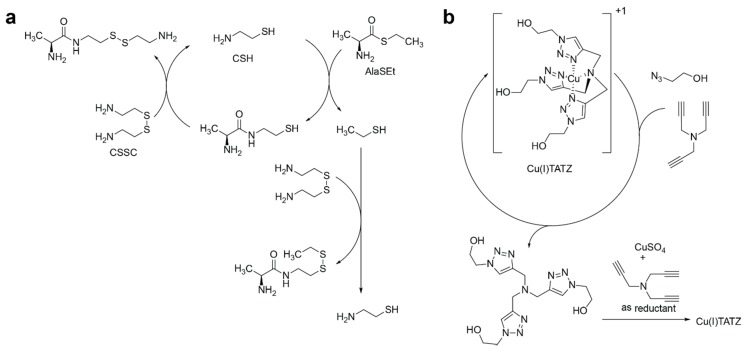
Schemes that represent (**a**) the thiol-based autocatalytic reaction and (**b**) the copper-catalyzed azide-alkine cycloaddition-based autocatalytic reaction as a catalytic cycle coupled to a non-catalytic reaction that converts one of the products of catalytic transformation into the catalyst itself. Examples of autocatalysts are cysteamine (CSH) and a copper complex (Cu(I)TATZ).

Semenov, Whitesides, and coworkers have recently published two autocatalytic reactions that can be reduced to a catalytic cycle, followed by the noncatalytic conversion of one product of this catalytic cycle to the catalyst itself (Figure 1) [37,38]. 

In the first example, we can separate a catalytic cycle of cysteamine (CSH), which catalyzed the acylation of cystamine (CSSC) by a thioester; this is followed by converting one of its byproducts, ethanethiol, into CSH (Figure 1a) [37]. In the second example, the catalytic cycle of the copper-catalyzed azide-alkyne cycloaddition reactions is followed by the formation of a catalytically active complex from triazole derivatives (Figure 1b) [38]. Because of the abundance of catalytic reactions in nature, we speculate that these motifs might be among the most common autocatalytic motifs in simple (i.e., non-biological) reaction networks.

**Figure 2:**
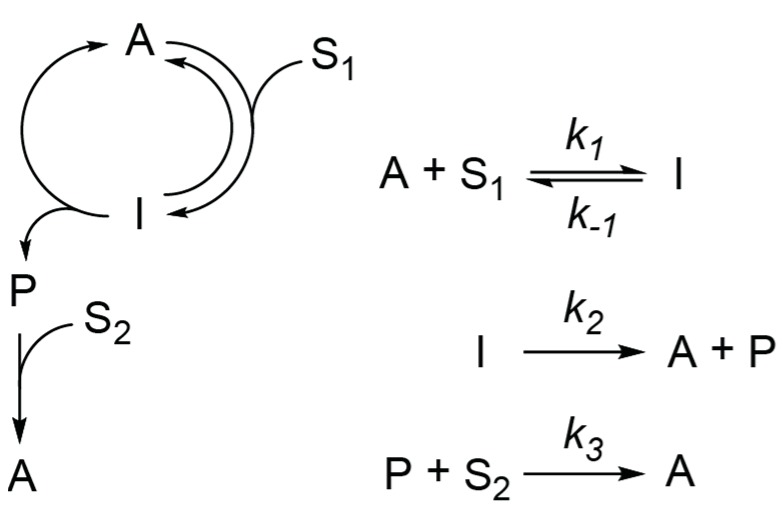
The autocatalytic reaction network analyzed here.

Here, we would like to analyze the kinetic behavior of one type of these motifs where the Michaelis-Menten-type catalysis is coupled to one additional irreversible reaction (Figure 2). 

We decided to analyze this particular motif because the Michaelis-Menten scheme can be applied to many catalytic reactions and possibility to reduce extended motifs to this motif by considering only rate-limiting steps. We would like to determine under which conditions this reaction network can be used in place of quadratic autocatalysis in experiments investigating chemical evolution and chemical systems with nonlinear kinetics and whether the kinetics of this network will always cause mutual exclusion of competing replicators.

## 2. Results and Discussion 

### 2.1. Analysis of Kinetics for a Network with an Infinite Supply of Substrates

Equations (1)–(3) with constant S_1_ and S_2_ describe the network in this approximation:

(1) $$\frac{dA}{dt}=-k_{1}S_{1}A+(k_{2}+k_{-1})I+k_{3}S_{2}P$$

(2) $$\frac{dI}{dt}=k_{1}S_{1}A-(k_{2}+k_{-1})I$$

(3) $$\frac{dP}{dt}=k_{2}I-k_{3}S_{2}P$$

Let us first consider two of the simplest cases: (i) when quasi-steady-state approximation (QSSA) can be applied to (dIdt=0) and P (dPdt=0) and (ii) when QSSA can be applied to A (dAdt=0) and P (dPdt=0). Simple calculations (see the Appendix A) show that in the first case the reaction is perfectly autocatalytic with the effective rate constant *k_e_* = *k_1_S_1_k_2_/(k_2_ + k_−1_)*:

(4) $$\frac{dA}{dt}=\frac{k_{1}S_{1}k_{2}}{\left(k_{2}+k_{-1}\right)}A$$

Obviously, in a situation with a limited amount of S_1_, the reaction will behave as quadratic autocatalysis with rate constant *k_e_*.

In the second case, the reaction is entirely autocatalytic for I with *k*_2_ as the autocatalytic constant (see the Appendix A):

(5) $$\frac{dI}{dt}=k_{2}I$$

Let us next look at a situation where we apply QSSA only to I. This situation is important for experimental systems because it has been shown that many catalytic reactions follow Michaelis –Menten kinetics, which implies QSSA for I. The Equations (1)–(3) can be reduced to the second order Equation (6) on P:

(6) $$\frac{d^{2}P}{dt^{2}}+k_{3}S_{2}\frac{dP}{dt}-\frac{k_{3}S_{2}k_{1}S_{1}k_{2}}{\left(k_{2}+k_{-1}\right)}P=0$$

From 6, with initial conditions P(0) = 0 and A(0) = A_0_, we can derive an expression for A (see the Appendix A for details).

(7) $$\begin{array}{l} A(t)=\frac{A_{0}}{\sqrt{k_{3}S_{2}(k_{3}S_{2}+4k_{1}S_{1}k_{2}/(k_{2}+k_{-1}))}}((k_{3}S_{2}+\lambda_{1})e^{\lambda_{1}t}-(k_{3}S_{2}+\lambda_{2})e^{\lambda_{2}t})\\ \lambda_{1/2}=\frac{-k_{3}S_{2}\pm \sqrt{k_{3}S_{2}(k_{3}S_{2}+4k_{1}S_{1}k_{2}/(k_{2}+k_{-1}))}}{2} \end{array}$$

Note that for physical (positive) rate constants and concentrations, λ_1_ is always positive and λ_2_ is always negative. The first term in Equation (7) has a positive exponent (λ_1_) and has the higher coefficient in front of the exponent other than the second term, and therefore, it will dominate from the beginning. Chemically, this equation means that if intermediates of the catalytic cycle do not accumulate in significant amounts, the reaction will behave as exponential autocatalysis from the beginning of the experiment and any deviations from exponential growth will decay over time.

Next, we will examine a situation where we do not have an accumulated product of the catalytic cycle because of the substantially high rate of its conversion to the autocatalyst. This situation is described by QSSA with dPdt=0 and cannot be reduced to the second order differential equation; instead, we need to solve a system involving two equations:

(8) $$\frac{dA}{dt}=-k_{1}S_{1}A+(2k_{2}+k_{-1})I$$

(9) $$\frac{dI}{dt}=k_{1}S_{1}A-(k_{2}+k_{-1})I$$

For the initial conditions, A(0) = A_0_ and I(0) = 0; this system has a solution:

(10) $$\begin{array}{l} A(t)=A_{0}(C_{1}e^{\lambda_{1}t}+C_{2}e^{\lambda_{2}t})\\ \lambda_{1/2}=\frac{-k_{1}S_{1}-k_{2}-k_{-1}\pm \sqrt{{\left(k_{1}S_{1}+k_{2}+k_{-1}\right)}^{2}+4k_{1}Sk_{2}}}{2} \end{array}$$

Both the *C*_1_ and *C*_2_ coefficients are positive and *C*_1_ + *C*_2_ = 1 (for an exact expression of *C*_1_ and *C*_2_, see the Appendix A). The value λ_1_ is always positive and λ_2_ is always negative for any positive rate constants. Therefore, the value of the decaying term, $A_{0}C_{2}e^{\lambda_{2}t}$, will not exceed that of *A*_0_. These calculations indicate that if in an experimental system the product P does not accumulate and *A*_0_ is much smaller than the concentration of the substrate, S_1_, this system will behave as an almost perfect quadratic autocatalysis.

Finally, we will briefly examine a situation where we do not apply any QSSA. For an autocatalyst A, the solution has a general form:

(11) $$A(t)=C_{1}e^{\lambda_{1}t}+C_{2}e^{\lambda_{2}t}+C_{3}e^{\lambda_{3}t}$$

If any part of λ_1-3_ is positive, then A will grow exponentially after an initial lag period (the term with a positive exponent will dominate in Equation (11)). As we show in the Appendix A, because rate constants are positive, one of the λ_1-3_ values must be positive. Thus, independently of rate constants and after some lag period, this reaction network will produce exponential growth. Experimental systems are limited by the amounts of the substrates S_1_ and S_2_ and might not have time to reach an exponential phase, especially with a high initial concentration of A.

### 2.2. Competition of the Autocatalysts of Two Different Autocatalytic Networks for Common Substrates

To describe a practically interesting situation, we analyzed the competition between two autocatalytic networks in a continuously stirred tank reactor (CSTR). Network 1 consists of A_1_, I_1_, and P_1_ and network 2 consists of A_2_, I_2_, and P_2_; they compete for common substrates S_1_ and S_2_. Here, the system is defined by:

(12) $$\frac{dA_{1}}{dt}=-k_{1}S_{1}A_{1}+(k_{2}+k_{-1})I_{1}+k_{3}S_{2}P_{1}-k_{0}A_{1}$$

(13) $$\frac{dI_{1}}{dt}=k_{1}S_{1}A_{1}-(k_{2}+k_{-1}+k_{0})I_{1}$$

(14) $$\frac{dP_{1}}{dt}=k_{2}I_{1}-(k_{3}S_{2}+k_{0})P_{1}$$

(15) $$\frac{dA_{2}}{dt}=-k_{1}^{\prime }S_{1}A_{2}+(k_{2}^{\prime }+k_{-1}^{\prime })I_{2}+k_{3}^{\prime }S_{2}P_{2}-k_{0}A_{2}$$

(16) $$\frac{dI_{2}}{dt}=k_{1}^{\prime }S_{1}A_{2}-(k_{2}^{\prime }+k_{-1}^{\prime }+k_{0})I_{2}$$

(17) $$\frac{dP_{2}}{dt}=k_{2}^{\prime }I_{1}-(k_{3}^{\prime }S_{2}+k_{0})P_{2}$$

(18) $$\frac{dS_{1}}{dt}=-(k_{1}A_{1}+k_{1}^{\prime }A_{2}+k_{0})S_{1}+k_{-1}I_{1}+k_{-1}^{\prime }I_{2}+k_{0}S_{10}$$

(19) $$\frac{dS_{2}}{dt}=-(k_{3}P_{1}+k_{3}^{\prime }P_{2}+k_{0})S_{2}+k_{0}S_{20},$$

where S_10_ and S_20_ are the concentrations at which S_1_ and S_2_ are supplied to the reactor. 

We should mention at this point that if networks compete only for substrate S_1_ and S_2_ is considered to be in an unlimited supply (i.e., we consider Equations (12)–(18) with S_2_ being constant), autocatalysts A_1_ and A_2_ cannot coexist at a steady state if any difference between the rate constants of the reactions of the networks exists (see the Appendix A). The model with constant S_2_ describes experimental systems where S_2_ is in a big excess in relation to S_1_ or where conversion of P to A is a monomolecular reaction. 

**Figure 3:**
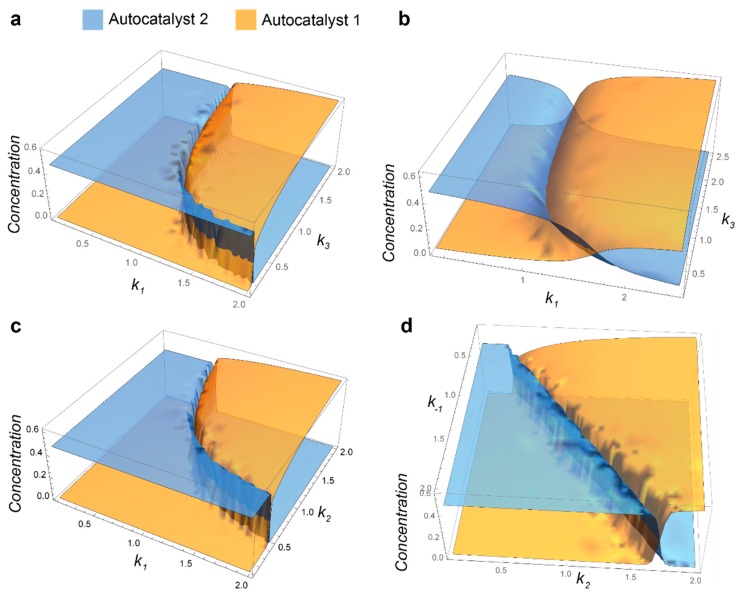
Competition between replicators A_1_ and A_2_ in continuously stirred tank reactor (CSTR). (**a**) The system is based on Equations (12)–(19). All reaction rate constants except k_1_ and k_3_ are set to 1; k_0_ is set to 0.1, S_10_ and S_20_ are set to 1, and k_1_ and k_3_ are varied from 0.1 to 2. (**b**) When considering the noncatalytic formation of A_1_ and A_2_ with k_4_ = k_4_′ = 0.01, other parameters are identical to *a*. (**c**) The system is based on Equations (12)–(19). All reaction rate constants except k_1_ and k_2_ are set to 1; k_0_ is set to 0.1, S_10_ and S_20_ are set to 1, and k_1_ and k_2_ are varied from 0.1 to 2. (**d**) The system is based on Equations (12)–(19). All reaction rate constants except k_−1_ and k_2_ are set to 1; k_0_ is set to 0.1, S_10_ and S_20_ are set to 1, and k−_1_ and k_2_ are varied from 0.1 to 2. In all plots, the concentrations of replicators A_1_ and A_2_ are plotted at t = 2000, A_1_(0) = 0.001, A_2_(0) = 0.001.

If S_2_ is variable, we cannot draw a simple conclusion about coexistence and need to use numerical analysis. We analyzed 12–19 using Mathematica script (see the Appendix A). Figure 3a shows concentrations of A_1_ and A_2_ at t = 2000 where, in most cases, the system reaches a steady state. We set all constants to unity, the initial concentrations of A_1_ and A_2_ to 0.001, *k*_0_ to 0.1, and varied *k*_1_ and *k*_3_, which characterize reactions with substrates. The graph has two characteristic futures: (i) autocatalysts A_1_ and A_2_ do not coexist, if one has a nonzero concentration and another falls to zero; (ii) competition is not sensitive to *k*_3_ as soon as *k*_3_ is sufficiently high. These features mean that these networks can undergo Darwinian evolution and that this evolution will be more sensitive to improvements in *k*_1_ than in *k*_3_. 

An important difference between model 12–19 and plausible chemical systems is the presence of the noncatalytic conversion of substrates to autocatalysts in many experimental systems. To consider these reactions, we have to add $k_{4}S_{1}$ and $k_{4}^{\prime }S_{1}$ to Equations (12) and (13) correspondingly, and subtract both these terms from Equation (18). We performed a numerical analysis of the modified equations with the same parameters as in Figure 3a and with $k_{4}$ and $k_{4}^{\prime }$ set to 0.01 (Figure 3b). The graph shows that autocatalysts can coexist and the region of coexistence is higher for low k_3_ values. Thus, we should avoid accumulating P in experimental systems with noncatalytic background reactions. We also explored how *k*_2_ and *k*_−1_ influence the competition between two replicators (Figure 3c,d). The plots demonstrate that *k*_2_, which represents *k_cat_* in a classical Michaelis-Menten scheme, has a stronger influence on the competition than *k*_3_ does, but it is less important than *k*_1_. Interestingly, simultaneously varying *k*_2_ and *k*_−1_ produces almost a symmetrical plot, which indicates their equal contribution to the competition between replicators (Figure 3d). Overall, the data indicate that *k*_1_, which is responsible for initiating the catalytic cycle, has the greatest effect on the competition, whereas *k*_3_, which is responsible for an axillary reaction that produces an extra molecule in the catalyst, has a minimum effect.

## 3. Conclusions

In this work, we determined, as precisely as possible, where and how to use reactions in experimental systems, which are based on the scheme shown in Figure 2. The results provide two main conclusions: (i) As soon as an intermediate of catalytic I or an intermediate product P does not accumulate in the reaction (at least one of them can be described by QSSA), the reaction kinetics does not deviate from exponential autocatalysis after a short lag period. (ii) The competition between these networks results in the mutual exclusion of autocatalysts if the noncatalytic formation of autocatalysts is negligible. Therefore, although these networks are not direct autocatalysts from a mechanistic perspective, they will behave as simple quadratic autocatalysts in most experimental systems.

Finally, if some variable information in autocatalyst A is transferred to product P and then retained during conversion of P to A, the network fulfills the conditions for Darwinian evolution. Interestingly, an experimental system from Otto’s group is [39,40], in a way, already following this mechanism. The growth of the supramolecular stacks, which is catalyzed by the terminus of the stack, is the catalytic step with information transfer; breaking the stack, which generates an extra terminus, is a P to A step that retains the information.

## Appendix A

- Equations (2)–(3) at dIdt=0 and dPdt=0 give:
  (A1) $$k_{1}S_{1}A-(k_{2}+k_{-1})I=0$$
  (A2) $$k_{2}I-k_{3}S_{2}P=0$$
  (A3) $$I=\frac{k_{1}S_{1}A}{k_{2}+k_{-1}}$$
  (A4) $$P=\frac{k_{2}k_{1}S_{1}A}{k_{3}S_{2}(k_{2}+k_{-1})}$$
  Substitution of (A3) and (A4) into (1) gives Equation (4)
  Equations (1) and (3) at dAdt=0 and dPdt=0) give:
  (A5) $$-k_{1}S_{1}A+(k_{2}+k_{-1})I+k_{3}S_{2}P=0$$
  (A6) $$P=\frac{k_{2}I}{k_{3}S_{2}}$$
  (A7) $$A=-\frac{(2k_{2}+k_{-1})I}{k_{1}S_{1}}$$
  Substitution of (A7) into (2) gives Equation (5)
- Equation (6) at P(0) = 0 and A(0) = A_0_ => dPdt(0)=k1S1k2(k2+k−1)A0 has a solution:
  (A8) $$\begin{array}{l} P(t)=\frac{A_{0}k_{1}S_{1}k_{2}}{(k_{2}+k_{-1})\sqrt{k_{3}S_{2}(k_{3}S_{2}+4k_{1}S_{1}k_{2}/(k_{2}+k_{-1}))}}(e^{\lambda_{1}t}-e^{\lambda_{2}t});\\ \lambda_{1/2}=\frac{-k_{3}S_{2}\pm \sqrt{k_{3}S_{2}(k_{3}S_{2}+4k_{1}S_{1}k_{2}/(k_{2}+k_{-1}))}}{2} \end{array}$$
  Considering that:
  (A9) $$A(t)=\frac{\left(k_{2}+k_{-1}\right)}{k_{1}S_{1}k_{2}}(k_{3}S_{2}P+\frac{dP}{dt})$$
  A(t) is described by Equation (7).
- In Equation (10) coefficients C_1_ and C_2_ are expressed by:
  (A10) $$\begin{array}{l} C_{1}=\frac{k_{1}S_{1}-k_{2}-k_{-1}+\sqrt{{\left(k_{1}S_{1}+k_{2}+k_{-1}\right)}^{2}+4k_{1}S_{1}k_{2}}}{2\sqrt{{\left(k_{1}S_{1}+k_{2}+k_{-1}\right)}^{2}+4k_{1}S_{1}k_{2}}}\\ C_{2}=\frac{k_{2}+k_{-1}-k_{1}S_{1}+\sqrt{{\left(k_{1}S_{1}+k_{2}+k_{-1}\right)}^{2}+4k_{1}S_{1}k_{2}}}{2\sqrt{{\left(k_{1}S_{1}+k_{2}+k_{-1}\right)}^{2}+4k_{1}S_{1}k_{2}}} \end{array}$$
- Equations (1)–(3) are linear and a solution for this linear system of ODE has the following form:
  (A11) $$\left(\begin{array}{c} A\\ I\\ P \end{array}\right)=C_{1}e^{\lambda_{1}t}{\tilde{\nu }}_{1}+C_{2}e^{\lambda_{2}t}{\tilde{\nu }}_{2}+C_{3}e^{\lambda_{3}t}{\tilde{\nu }}_{3}$$
  where λ_1_, λ_2_, and λ_3_ are eigenvalues, and ν_1-3_ are the corresponding eigenvectors of the matrix:
  (A12) $$\left(\begin{array}{ccc} -k_{1}S_{1} & k_{2}+k_{-1} & k_{3}S_{2}\\ k_{1}S_{1} & -k_{2}-k_{-1} & 0\\ 0 & k_{2} & -k_{3}S_{2} \end{array}\right)$$
  λ_1_, λ_2_, and λ_3_ are roots of the equation:
  (A13) $$\lambda^{3}+(k_{1}S_{1}+k_{2}+k_{-1}+k_{3}S_{2})\lambda^{2}+k_{3}S_{2}(k_{1}S_{1}+k_{2}+k_{-1})\lambda -k_{1}S_{1}k_{3}S_{2}k_{2}=0$$
  From Vieta’s formula:
  (A14) $$\lambda_{1}\lambda_{2}\lambda_{3}=k_{1}S_{1}k_{3}S_{2}k_{2}$$
  Because *k*_1_, *k*_2_, *k*_3_, *S*_1_, *S*_2_ are positive, one of the λ_1-3_ values must be positive.
- Steady-state conditions for 12-19 are as follows:
  (A15) $$A_{1}(-k_{1}S_{1}+\frac{(k_{2}+k_{-1})k_{1}S_{1}}{k_{2}+k_{-1}+k_{0}}+\frac{k_{2}k_{1}S_{1}k_{3}S_{2}}{\left(k_{3}S_{2}+k_{0})(k_{2}+k_{-1}+k_{0}\right)}-k_{0})=0$$
  (A16) $$A_{2}(-{k^{\prime }}_{1}S_{1}+\frac{({k^{\prime }}_{2}+{k^{\prime }}_{-1}){k^{\prime }}_{1}S_{1}}{{k^{\prime }}_{2}+{k^{\prime }}_{-1}+k_{0}}+\frac{{k^{\prime }}_{2}{k^{\prime }}_{1}S_{1}{k^{\prime }}_{3}S_{2}}{\left({k^{\prime }}_{3}S_{2}+k_{0})({k^{\prime }}_{2}+{k^{\prime }}_{-1}+k_{0}\right)}-k_{0})=0$$
  If we consider S_2_ to be constant and the reaction rates are different for two networks, this system of equations does not have solutions where both A_1_ and A_2_ are nonzero.
- Mathematic script that generates the plot 3A. Plots 3B-C were generated by modifying this script.
  k0 = 0.1; k1 = 1; k1r = 1; k2 = 1; k3 = 1; k1z = 1; k1rz = 1; k2z = 1; k3z = 1; S01 = 1; S02 = 1;
  s = ParametricNDSolve[{a’[t] == -a[t]*(i*k1*s1[t] + k0) + b[t]*(k2 + k1r) + h*k3*s2[t]*p[t], b’[t] == i*k1*a[t]*s1[t] - b[t]*(k2 + k1r + k0), p’[t] == k2*b[t] - (h*k3*s2[t] + k0)*p[t], a1’[t] == -a1[t]*(k1z*s1[t] + k0) + b1[t]*(k2z + k1rz) + k3z*s2[t]*p1[t], b1’[t] == k1z*a1[t]*s1[t] - b1[t]*(k2z + k1rz + k0), p1’[t] == k2z*b1[t] - (k3z*s2[t] + k0)*p1[t], s1’[t] == -i*k1*a[t]*s1[t] - k1z*a1[t]*s1[t] + k1r*b[t] + k1rz*b1[t] - k0*s1[t] + k0*S01, s2’[t] == -h*k3*p[t]*s2[t] - k3z*p1[t]*s2[t] - k0*s2[t] + k0*S02, a[0] == 0.001, a1[0] == 0.001, b[0] == p[0] == b1[0] == p1[0] == 0, s1[0] == s2[0] == 1 }, {a, b, p, a1, b1, p1, s1, s2}, {t, 0, 2000}, {i, h}];
  Plot3D[{a[i, h][2000] /. s, a1[i, h][2000] /. s}, {i, 0.1, 2}, {h, 0.1, 2}, PlotStyle -> Opacity[0.7], Mesh -> None, AxesStyle -> 16]
